# Anti-tumor Necrosis Factor Alpha Versus Corticosteroids: A 3-fold Difference in the Occurrence of Venous Thromboembolism in Inflammatory Bowel Disease－A Systematic Review and Meta-analysis

**DOI:** 10.1093/ecco-jcc/jjad193

**Published:** 2023-11-11

**Authors:** Hajnal Székely, Laura Mária Tóth, Anett Rancz, Anna Walter, Nelli Farkas, Miklós Domonkos Sárközi, Szilárd Váncsa, Bálint Erőss, Péter Hegyi, Pál Miheller

**Affiliations:** Department of Surgery, Transplantation and Gastroenterology, Semmelweis University, Budapest, Hungary; Centre for Translational Medicine, Semmelweis University, Budapest, Hungary; Centre for Translational Medicine, Semmelweis University, Budapest, Hungary; Centre for Translational Medicine, Semmelweis University, Budapest, Hungary; Department of Internal Medicine and Hematology, Medical School, Semmelweis University, Budapest, Hungary; Centre for Translational Medicine, Semmelweis University, Budapest, Hungary; Institute of Bioanalysis, Medical School, University of Pécs, Pécs, Hungary; General Medicine, Faculty of Medicine, Semmelweis University, Budapest, Hungary; Centre for Translational Medicine, Semmelweis University, Budapest, Hungary; Institute for Translational Medicine, Medical School, University of Pécs, Pécs, Hungary; Institute of Pancreatic Diseases, Semmelweis University, Budapest, Hungary; Centre for Translational Medicine, Semmelweis University, Budapest, Hungary; Institute for Translational Medicine, Medical School, University of Pécs, Pécs, Hungary; Institute of Pancreatic Diseases, Semmelweis University, Budapest, Hungary; Centre for Translational Medicine, Semmelweis University, Budapest, Hungary; Institute for Translational Medicine, Medical School, University of Pécs, Pécs, Hungary; Institute of Pancreatic Diseases, Semmelweis University, Budapest, Hungary; Department of Surgery, Transplantation and Gastroenterology, Semmelweis University, Budapest, Hungary

**Keywords:** Complication, biologics, conventional therapy

## Abstract

**Background and Aims:**

Patients with inflammatory bowel disease [IBD] have a more than two fold higher risk of venous thromboembolic events [VTE] than the general population. The aetiology is complex, and the role of medication is not precisely defined. We aimed to assess the effects of anti-tumor necrosis factor alpha [anti-TNFα] drugs and conventional anti-inflammatory therapy, namely corticosteroids [CS], immunomodulators [IM], and 5-aminosalicylates [5-ASA] on VTE in IBD.

**Methods:**

A systematic search was performed in five databases on November 22, 2022. We included studies reporting VTE in the distinct categories of medications, determined the proportions, and calculated the odds ratios [OR] with 95% confidence intervals [CI], using the random-effects model. The risk of bias was evaluated with the Joanna Briggs Institute Critical Appraisal Checklist and the Risk of Bias in Non-randomized Studies of Interventions tool.

**Results:**

The quantitative analysis included 16 observational studies, with data from 91 322 IBD patients. Patients receiving anti-TNFα medication had significantly less VTE [proportion: 0.05, CI: 0.02–0.10], than patients treated with CS [proportion: 0.16, CI: 0.07–0.32], with OR = 0.42 [CI: 0.25–0.71]. IMs resulted in similar proportions of VTE compared with biologics [0.05, CI: 0.03–0.10], with OR = 0.94 [CI: 0.67–1.33]. The proportion of patients receiving 5-ASA having VTE was 0.09 [CI: 0.04–0.20], with OR = 1.00 [CI: 0.61–1.62].

**Conclusions:**

Biologics should be preferred over corticosteroids in cases of severe flare-ups and multiple VTE risk factors, as they are associated with reduced odds of these complications. Further studies are needed to validate our data.

## Graphical Abstract

**Graphical Abstract:**
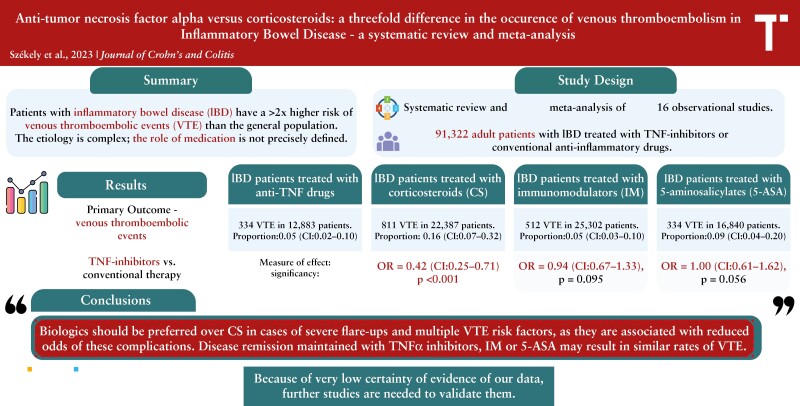

## 1. Introduction

Patients with inflammatory bowel disease [IBD] have an approximately two to three times higher risk of venous thromboembolic events [VTE] compared with non-IBD controls.^1–3^ The incidence is significantly higher [7.6% vs 3.3%, *p* <0.0001] than in age-and sex-matched controls.^4^ The prevalence varies between 1.2 and 6.7% [up to 39% in postmortem studies].^5^ It affects patients of both sexes^1,2,4^ with Crohn’s disease [CD] and ulcerative colitis [UC].

The clinical features of VTE in IBD are well known. It presents at a younger age,^4,6^ with high rates during disease remission (hazard ratio [HR]: 2.1; CI: 1.6–2.9) and acute flare-ups [HR: 8.4; CI: 5.5–12.8].^5,7^ The risk also increases in the outpatient setting^7–9^ and in the postoperative period,^10,11^ but it is considered to be the highest during periods of active disease flare-ups and prolonged hospitalisation.^4,7,12–16^

It is also characterised by high recurrence rates^4,17^ and poor prognosis. The mortality rate is about two and a half times higher compared with IBD patients without VTE^4^ and more than twice as high compared with age- and comorbidity-adjusted non-IBD patients,^13^ reaching 22%.^18^

IBD itself is considered to be an independent VTE risk factor.^5^ Disease- and treatment-specific characteristics include extensive colitis in UC, colonic involvement and fistulizing behaviour in CD, active disease flare-ups with consequent hospitalisation, intravenous corticosteroid [CS] therapy, and surgery.^5,7,10,13,15,19^

The pathogenesis of VTE in IBD is complex, and chronic inflammation is hypothesised to play a pivotal role. It triggers pro-coagulation phenomena, interferes with the fibrinolytic system, and activates platelets and endothelial cells, affecting cell adhesion molecules and inflammatory cytokines.^20,21^ Tumor necrosis factor alpha [TNFα] may also have a role. Activation of the intrinsic coagulation pathways through the expression of tissue factor and cell-adhesion molecules^21,22^ and the interaction with other inflammatory cytokines result in the downregulation of natural anticoagulant processes, increased platelet production, and the promotion of thrombin formation. It also acts on the endothelial surface of blood vessels.^22^

The VTE risk in IBD patients should be individually estimated, as it may interfere with the optimal medical treatment strategy. As the role of anti-inflammatory medication and the magnitude of its effect are still a matter of debate, we aimed to calculate the proportion and odds of VTE in IBD patients treated with anti-TNFα agents and conventional drugs (CS, immunomodulators [IM], 5-aminosalicylates [5-ASA]), with the hypothesis that biologics are associated with the reduced odds of these events.

## 2. Materials and Methods

We adhered to the recommendations of the Cochrane Collaboration^23^ and reported our work on the Preferred Reporting Items for Systematic Reviews and Meta-analyses [PRISMA] guidelines.^24^ [Supplementary Material, Table 1]. Details are available on the International Prospective Register of Systematic Reviews [PROSPERO] surface [registration number CRD42022376118].

### 2.1. Information sources and search strategy

A comprehensive literature search was conducted in five electronic databases: Medline [via PubMed], Embase, Cochrane Central Register of Controlled Trials [CENTRAL], Scopus, and Web of Science. Our search key containing the domains of the disease, the complication, and the chosen biologic drugs, with the medical subject headings [MeSH] and free-text terms, is available in the Supplementary Material (1. Search Key). No filters or restrictions were applied besides the TITLE-ABS-KEY filter in the Scopus database.

Data were collected from inception to November 22, 2022. Reference lists and citations of relevant articles were scanned on February 22, 2023.^25^ In the case of missing data, authors of eligible articles were contacted.

### 2.2. Eligibility criteria

We included observational population-based data, cohort- and case-control studies. Case series, case reports, and conference abstracts, as well as studies of paediatric patients [age <18 years] were excluded. We used the 'PICO' framework for the research question and to identify eligible studies. P [population] consists of adult patients [>18 years] with IBD, and I [intervention] represents the anti-TNFα therapy, with C [comparison] implying conventional anti-inflammatory treatment [CS, IM, 5-ASA]. O [outcome] comprises clinical manifestations of VTE [deep vein thrombosis, pulmonary embolism, porto-mesenteric vein thrombosis, cerebral venous sinus thrombosis, and central venous catheter-related events].

Studies reporting the number of VTE in patients treated with anti-TNFα drugs, CS, IM, or 5-ASA during hospitalisation or as outpatients were selected for the quantitative analysis. Measures that could not be pooled were qualitatively synthesised. When available, data were collected separately for the two disease phenotypes. As a secondary outcome, we planned to collect data on mortality of patients with VTE. We planned to exclude patients with active malignancies and ongoing anticoagulation.

### 2.3. Study screening and selection

After extracting relevant records, these were exported to EndNote^TM^20 bibliographic management software,^26^ where duplicates were automatically and then manually removed. References were uploaded, and study selection was performed on the Rayyan platform^27^ first by title and abstract, then by full text by two independent authors [HSz, LMT], with the level of agreement measured by Cohen’s kappa coefficient.^28^

References of the selected articles and citation searching were conducted using the CitationChaser tool,^25^ followed by the selection of eligible papers on the Rayyan application,^27^ which was performed independently by two reviewers [HSz, LMT]. The inter-rater reliability was determined by calculating Cohen’s kappa coefficient.^28^ A third independent author [MDS] resolved disagreements at each step.

### 2.4. Data collection

Two members of the team [HSz, LMT], as well as a third independent reviewer, extracted data separately: first author, year of publication, Digital Object Identifier [DOI], country of the study, period of the study, study design, single/multicentre study, and study population. The total number of IBD patients and patients with VTE were collected according to the reported medication [anti-TNFα or conventional anti-inflammatory therapy]. When available, these data were also assessed by disease phenotype. Characteristics of the IBD population [age, sex, smoking status, age at diagnosis, and disease duration], comorbidities, previous VTE, hospitalisation, immobilisation, and mortality were also extracted. The raw data of the authors^4,29^ and conversion of proportion^4^ were also used. Data were collected in a predesigned Excel spreadsheet [Office 365, Microsoft, Redmond, WA, USA].

### 2.5. Study quality and risk of bias

Quality evaluation was performed by two independent reviewers [HSz, and LMT]. The risk of bias for the proportion analysis was assessed using the Joanna Briggs Institute Critical Appraisal Tool for Prevalence Studies^30^ [Supplementary Material, Table 2]. A predefined sample size calculation formula^31^ helped us to determine the sample size adequacy [Supplementary Material, Formula 1]. For articles comparing the effects of the two interventions, we used The Risk Of Bias In Non-randomized Studies of Interventions [ROBINS-I] tool^32^ [Supplementary Material, Table 3].

### 2.6. Certainty of evidence

Two review authors [HSz, and LMT] independently evaluated the overall quality of evidence, based on the recommendations of the ‘Grading of Recommendation, Assessment, Development, and Evaluation’ [GRADE] Working Group.^33^ For the Summary of Findings table and evaluation of proportion tables, the GRADE profiler [GRADEpro] tool was used.^34^ Details are available in the Supplementary Material (GRADE assessment).

### 2.7. Data synthesis and analysis

To estimate the probability of VTE in IBD patients treated with anti-TNFα drugs compared with those receiving conventional anti-inflammatory therapy, an effect size measure odds ratio [OR] with a 95% confidence interval [CI] was calculated. Because of relevant between-study heterogeneity, a random-effects model was applied. Considering concomitant medication, we also determined the proportion of these events with 95% CI for more appropriate results, based on the total numbers of patients and of those with VTE in each medication group.

For subgroup analysis, we followed the descriptions of Harrer *et al*.^35^

As mortality of VTE was not reported in association with medication, we were not able to summarise these data. It was not possible to exclude patients with active malignancies or those on simultaneous anticoagulation, because of inadequate data on specific treatment categories.

The Mantel–Haenszel method was used for pooled OR based on 'raw data'.^36^ The Paule–Mandel model was applied to estimate the heterogeneity variance measure [τ2] for raw data OR calculation. For direct OR calculation, the restricted maximum-likelihood estimator, and for the confidence interval, the Q profile method, were used. Results were considered statistically significant if the CI did not contain the value 1.^37^

We assessed heterogeneity using the Higgins and Thompson I² statistics.^38^ I^2^ values representing moderate [30–60%], substantial [50–90%], and considerable [75–100%] heterogeneity were based on the Cochrane Collaboration recommendations.

Forest plots and regression plots were used to summarise the findings. We reported the prediction intervals [ie, the expected range of effects of future studies] of results.^39^ Publication bias was evaluated by visual assessment of the funnel plot and tests for funnel plot asymmetry using Egger’s tests.^40^

The R software [R Core Team, 2019, Vienna, Austria],^41^ meta,^42^ dmetar,^43^ and metafor**^44^** packages, were used for the statistical analysis.

## 3. Results

### 3.1. Study selection

**Figure 1:**
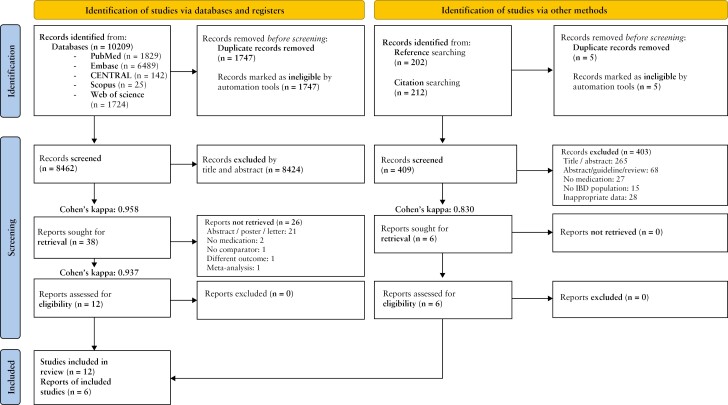
Prisma 2020 flowchart of the study selection process

Our search key identified 10 209 reports. Backward and forward citation searching of the included articles resulted in 202 and 212 studies, respectively. At the end of the selection process, 18 articles were found eligible, of which 16 were included in the quantitative synthesis [Figure 1].

### 3.2. Study characteristics

Sixteen studies were included in the meta-analysis,^4,14,29,45–57^ of which one was prospective,^46^ five were monocentric cohort studies,^14,29,47,54,55^ four were conducted in multiple centres,^46,50,52,53^ and three were population-based, nationwide cohort studies.^4,45,49^ One publication used public and private health insurance data,^50^ one was a real-world insurance claims analysis.^51^ Five papers used the case-control methodology to assess the role of risk factors [among them medication] of VTE in IBD.^4,48,53,56,57^

Two papers were considered for qualitative synthesis.^9,58^ The study by Ananthakrishnan *et al*.^9^ focused on VTE in the post-hospitalisation period, reporting the HR of these events in relation to medication. The paper by Kim *et al.*^58^ determined the adjusted HR [aHR] of VTE in accordance with anti-inflammatory drugs.

Out of 139 147 IBD patients, 91 322 were included in our quantitative synthesis. A total of 2381 patients had VTE. Data from 31 757 patients with CD and 39 832 patients with UC showed no relevant difference in the proportion of this complication between the two disease phenotypes [813 CD and 931 UC patients]. Demographic data of patients from included studies are presented in Table 1.

**Table 1:** Demographic data of the included studies.

Study’s first author, year of publication	Study design	Country	Study period	Nr. of IBD patients (CD/UC)	Nr. of VTE in IBD patients (CD/UC)	Nr. of male/female IBD patients with VTE	Age at VTE (years); Mean ± SD Median (IQR)	Disease duration (months/years) Mean ± SD Median (IQR)
**Quantitative synthesis**								
Alatri et al., 2016	Retrospective, nationwide cohort	Switzerland	2006–2013	2.284 (1.324/960)	90 (45/45)	46/44	CD: 50 (39–61) UC: 46 (40–59)	CD: 12 (8–23) years UC: 7 (4–18) years
Ando et al., prospective, 2018	Prospective, multicenter cohort	Japan	2013–2018	42 (20/22)	7 (1/6)	2/5	54.9 ± 21.3	40.1 ± 66.7 month
Ando et al., retrospective, 2018	Retrospective, monocentric cohort	Japan	2009–2013	340 (251/89)	24 (9/15)	14/10	49.0 ± 20.7	9.5 ± 10.1 years
Andrade et al., 2018	Retrospective, monocentric cohort	Brazil	2010–2015	1.093 (654/439)	56 (37/19)	24/32	NA	NA
Arora et al., 2016	Retrospective, monocentric case: control (1:3)	USA	2004–2013	80 (56/24)	20 (14/6)	11/9	44.9 ± 17.0	7.9 (1.6–29.4) years
Bernstein et al., 2021	Retrospective, population-based cohort + case: control (1:5)	Canada	1984–2018 + 2005–2018	11.262 (5.445/5.817)	854 (455/399)	373/481	NA	NA
Curtis et al., 2021	Retrospective, population-based cohort of UC patients	USA	2010–2015	6.366	2.335 (0/2335)	NA	NA	NA
deFonseka et al., 2016	Retrospective, monocentric cohort	USA	2002–2011	547 (364/183)	50 (31/19)	24/26	52 ± 13	14 ± 14 years
Desai et al., 2017	Retrospective, cohort - health insurances	USA	2000–2013	21.671 (A*: 14.491/A*: 7.180)	312 (NA/NA)	A*	A*	NA
Fujiya et al., 2022	Retrospective, cohort - medical database	Japan	2013–2018	16.273 (3.443/13.585)	215 (65/160)	142/73	NA	NA
Higgins et al., 2014	Retrospective, multicenter cohort	USA	2003–2009	15.100 (A*)	335 (NA/NA)	NA	A**	NA
Liu et al., 2021	Retrospective, multicenter cohort+ case: control (1:3)	China	2011–2016	8.459 (4.122/4337)	46 (16/24)***	26/20	46.3 ± 15.7	39.1 (5.3–58.5) months
McCurdy et al., 2019	Retrospective, monocentric cohort	Canada	2009–2016	2.161 (1.453/688)	47 (33/14)	27/20	51.5 ± 15.2	NA
Ohta et al., 2019	Retrospective, monocentric cohort	Japan	2015–2016	72 (30/42)	6 (1/5)	1/5	46.8 ± 10.3	70.2 ± 68.07 months
Scoville et al., 2014	Retrospective, monocentric, case: control (1:1)	USA	2002–2012	204 (96/108)	204 (96/108)	98/106	54 ± 19	13 ± 14 years
Shujun et al., 2021	Retrospective, monocentric case: control (1:4)	China	2013–2020	5.368 (NA/NA)	26 (6/20)	17/9	34.9 ± 15	4 (2-6.9) years
**Qualitative analysis**								
Ananthakrishnan et al., 2014	Retrospective, multicenter cohort	USA	1994–2012	2.788	62 (NA/NA)	NA	A§	NA
Kim et al., 2022	Retrospective, nationwide cohort	Korea	2004 (2006)–2015	45.037 (13.850/31.187)	411 (106/305)	209/202	NA	NA

Abbreviations: CD, Crohn’s disease; IBD, inflammatory bowel disease; IQR, interquartile range; NA, not available; Nr., number; SD, standard deviation; UC, ulcerative colitis; VTE, venous thromboembolic event.

*Reported in percentages; **Reported in percentages related to medication; ***Demographic data available in 40 inpatients out of a total of 46 patients; §Reported according to in/outpatients status.

Although disease activity was not uniformly reported, most VTE cases presented in patients with active disease.^14,29,46–48,53,54,57^ Colon/ileocolonic involvement was most frequently assessed in patients with CD,^29,45,47,53^ stricturing or fistulizing disease behaviour was most common.^29,48,53^ In patients with UC, pancolitis was considered a disease-specific risk factor.^45,47,48,53^

Surgery increased the probability of this complication^45,51–53^; the paper published by Higgins *et al*.^52^ reported the most prominent effect [OR = 3.58, CI: 2.64–4.87].

Numerous comorbidities were assessed by the authors^4,14,46,49–54,57,58^: congestive heart failure [CHF], chronic obstructive pulmonary disease, acute coronary syndrome, diabetes mellitus, obesity, atrial fibrillation, peripheral vascular disease, cerebrovascular disease, chronic kidney disease [CKD], liver disease, and neuromuscular disease, using different measures. The most pronounced impact was published by Kim *et al.*^58^ for CKD [aHR = 3.36, CI: 1.24–9.14] and by Higgins *et al*.^52^ for CHF [OR = 3.91, CI: 0.89–17.25]. Cancer history was reported by some of the authors.^4,29,51–53^

Inpatient status, longer hospitalisation, immobilisation, and central venous catheter insertion were also considered to interfere with the VTE risk.^4,14,29,46–48,51–55,58^

Patients with IBD with this complication were more often smokers,^45,48,50,53,57^ and some of them had a history of VTE.^14,51,54,57^ Antithrombotic medication was rarely recorded.^9,14,29,46,54^

### 3.3. Proportion of VTE in IBD patients in different treatment groups

#### 3.3.1. Proportion of VTE in IBD patients treated with TNFα inhibitors

The number of VTE in patients treated with TNFα inhibitors was available from 16 articles. The summary of the data resulted in 334 VTEs in 12 883 IBD patients treated with biologic drugs,^4,14,29,45–57^ the proportion being 0.05 [CI: 0.02–0.10] [Supplementary Material, Figure 1].

Data for subgroup analysis of patients with CD and UC were available from five studies^4,29,45,51,53^ separately. In 2720 patients with CD and 1314 patients with UC, the proportion of VTE was 0.06 [CI: 0.02–0.21] and 0.09 [CI: 0.02–0.27], respectively [Supplementary Material, Figure 5A].

#### 3.3.2. Proportion of VTE in IBD patients treated with corticosteroids

The proportion of VTE in IBD patients treated with CS was calculated based on data from 13 articles. Among 22 387 patients, 811 VTE cases were reported [proportion: 0.16, CI: 0.07–0.32]^4,14,45–48,51–57^ [Supplementary Material, Figure 2].

Subgroup analysis by disease phenotype showed that CD patients treated with CS were more frequently affected by VTE than UC patients [proportion: 0.16 vs 0.13]^4,45,51,53^ [Supplementary Material, Figure 5B].

For the systematic review, two additional articles were considered. CS therapy significantly increased the VTE risk in the population-based cohort [aHR: 1.88, CI: 1.51–2.33, *p* <0.001]^58^ and also proved to be an independent predictor of post-hospitalisation VTE [HR: 1.71, CI: 1.02–2.87].^9^

#### 3.3.3. Proportion of VTE in IBD patients treated with immunomodulators

The sum of data from 13 articles^14,29,45–51,53–55,57^ resulted in a proportion of 0.05 [CI: 0.03–0.10] of the complication in patients treated with IMs [25 302 IBD patients with 512 VTE] [Supplementary Material, Figure 3].

CD and UC patients on IM medication were affected by VTE in similar proportions [0.06 and 0.09, respectively]^29,45,51,53^ [Supplementary Material, Figure 5C].

#### 3.3.4. Proportion of VTE in IBD patients treated with 5-aminosalicylates

Data on patients treated with 5-ASA drugs were extracted from 10 articles, involving 16 840 patients with IBD.^14,29,45,46,48,51,53,55–57^ A total of 334 had VTE [proportion: 0.09, CI: 0.04–0.20] [Supplementary Material, Figure 4].

Four articles reported data separately for patients with CD and UC.^29,45,51,53^ Of the 3430 patients with CD, 68 had VTE. Altogether, 153 events were identified in 13 315 patients with UC, with proportions of 0.06 [CI: 0.01–0.25] vs 0.04 [CI: 0.01–0.20] [Supplementary Material, Figure 5D].

### 3.4. Odds ratio of VTE in IBD patients treated with anti-TNFα and conventional anti-inflammatory therapy

#### 3.4.1. Odds of VTE in IBD patients treated with anti-TNFα and corticosteroids, respectively

**Figure 2:**
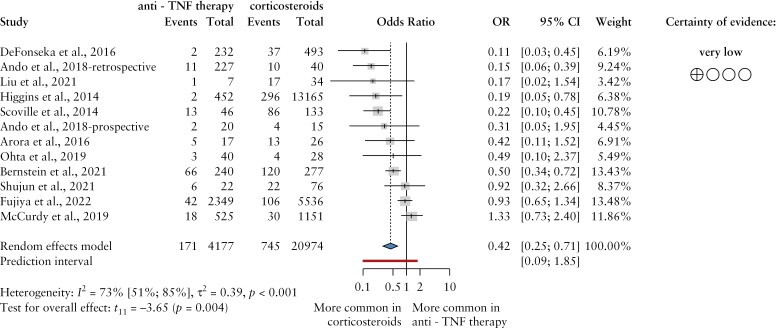
The odds ratio of venous thromboembolic events in patients with inflammatory bowel disease treated with anti-TNFα drugs and corticosteroids, respectively. Anti-TNF, anti-tumor necrosis factor; events, venous thromboembolic events; OR, odds ratio; CI, confidence interval.

Calculations confirmed an OR of VTE of 0.42 [CI: 0.25–0.71] in patients with IBD treated with anti-TNFα or CS, a statistically significant effect [*p* <0.001] [Figure 2]. The between-study heterogeneity was substantial [I^2^ = 73%, CI: 51–85%, *p* <0.001].

These data support that TNFα inhibitors are associated with lower probability [9% vs 16%] and odds of these events compared with CS therapy.

#### 3.4.2. Odds of VTE in IBD patients treated with anti-TNFα and immunomodulators, respectively

**Figure 3:**
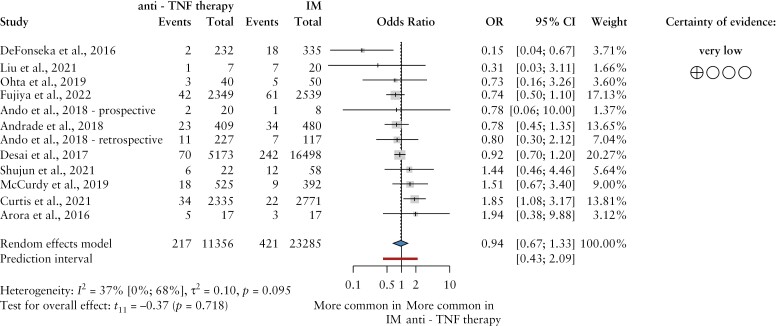
The odds ratio of venous thromboembolic events in patients with inflammatory bowel disease treated with anti-TNFα drugs and immunomodulators, respectively. Anti-TNF, anti-tumor necrosis factor; IM, immunomodulators; events, venous thromboembolic events; OR, odds ratio; CI, confidence interval.

The summary of data showed no difference in the probability of VTE in the two treatment groups [OR = 0.94, CI: 0.67–1.33] [Figure 3], with moderate study heterogeneity [I^2^ = 37%, CI: 0–68%].

#### 3.4.3. Odds ratio of VTE in IBD patients treated with anti-TNFα and 5-aminosalicylates, respectively

**Figure 4:**
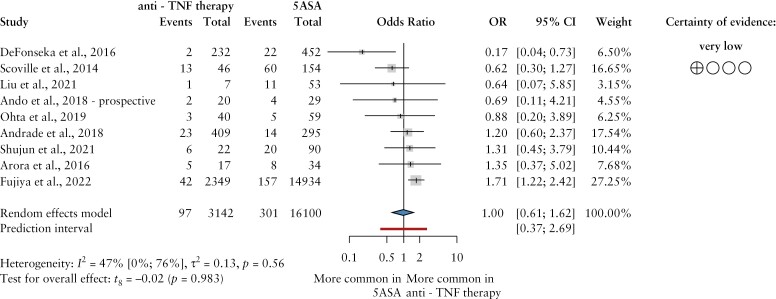
The odds ratio of venous thromboembolic events in patients with inflammatory bowel disease treated with anti-TNFα drugs and 5-aminosalicylates, respectively. Anti-TNF, anti-tumor necrosis factor; 5ASA, 5aminosalicylates; events, venous thromboembolic events; OR, odds ratio; CI, confidence interval.

The pooled data showed that patients receiving either anti-TNFα treatment or 5-ASA therapy were equally affected by VTE, as there was no difference in the odds of these events in the treatment categories mentioned [OR = 1.00, CI:0.61–1.62]. The I^2^ value of 47% [CI: 0–76%] proved moderate between-study heterogeneity [Figure 4].

##### 3.4.3.1. Risk of bias assessment

The assessment is presented in the Supplementary Material Tables 2 and 3. The studies are characterised by moderate to high levels of bias, primarily due to the numerous patient- and disease-specific confounding factors interfering with the occurrence of VTE. Reporting or adjusting for the most important ones－disease activity, location, CD behaviour, length of hospitalisation, central venous catheter, comorbidities, surgery, antithrombotic medication, malignant diseases, obesity, smoking, oral contraceptive drugs, and previous VTE－was incomplete. In some cases, the start of follow-up and the start of the intervention were inaccurately reported.

The studies by Fujiya *et al*.^51^ and Higgins *et al*.,^52^ as well as the two studies included in the qualitative synthesis,^9,58^ were considered to have a serious risk of bias due to lack of information on multiple important confounders. The study by Fujiya *et al*.^51^ did not report some of their data, contributing to bias in the selection of reported results.

Systematic heterogeneity across studies and publication bias were also estimated visually and presented in funnel plots [Supplementary Material, Figure 6A, B, C, D and Figure 7A, B, C] in each treatment category.

In the cases of studies included in our work, the asymmetry of one of the funnel plots [Supplementary Material, Figure 7A] suggests the possibility of systematic heterogeneity [clinical differences between studies] rather than publication bias. The symmetry of the other funnel plots suggests a low risk of publication bias.

##### 3.4.3.2. Certainty of evidence

We conclude that the overall certainty of evidence for our results is very low [Supplementary Material, Tables 4, 5, and 6]. The study design, the failure to adequately control confoundings, and inconsistency of data, as well as imprecision of results, were raised. A decisive aspect is the concomitant medication regimen characteristic for the IBD population, making it difficult to precisely assess the effect of each drug separately on VTE risk.

## 4. Discussion

Due to numerous factors, the aetiology of VTE in IBD is complex.

We would like to draw attention to this potentially fatal, preventable complication of IBD. We emphasise that a significant proportion of VTE may present without specific symptoms, underlining the need to summarise the risk factors of VTE in each patient and to assess their added effect. Individual risk should always be evaluated, as it may interfere with the optimal medical treatment strategy.

To determine the impact of the most commonly used anti-inflammatory medication on VTE risk, we pooled data on these events in adult IBD patients treated with anti-TNFα drugs and conventional therapy. The summary of these data showed a possible protective effect of anti-TNFα drugs compared with CS, as patients treated with these biologic drugs had an overall 58% reduction in VTE risk versus those on CS. There was no difference in the proportion or the odds of these events in patients treated with TNFα inhibitors and IM or 5-ASA.

Pooled data based on I^2^ values show significant heterogeneity; this is primarily attributable to confounders in the IBD population, for which studies were consequently not adjusting [eg, disease activity, localisation, disease behaviour in CD, hospitalisation/outpatient setting, comorbidities, malignant diseases, surgery, and thromboprophylaxis]. Differences in drug administration [dose, route, time interval, and precise temporal relationship with VTE] should be mentioned in CS-treated patients. Heterogeneity in IBD patients taking 5-ASA medication may also be encountered, as this constitutes the basic treatment option for UC patients and a proportion of CD patients, independent of the concomitant IBD therapy. Concurrent medication, which is a common practice in the IBD patient population, also interferes with the results. Differences in study design and follow-up time may also play a role.

A review of the literature shows that the effect of anti-inflammatory medication on VTE risk is not precisely defined. We highlight the importance of the first meta-analysis^59^ assessing the role of anti-TNFα and systemic corticosteroids on VTE in IBD patients. Data pooled from three studies showed that anti-TNFα treatment was associated with a significantly lower risk of VTE [OR = 0.26, CI: 0.106–0.674, *p* = 0.005].^9,14,52^ The beneficial effect of biologic agents was estimated by Higgins *et al*., reporting a nearly fivefold reduced risk of this complication compared with CS therapy [OR = 0.21, CI: 0.05–0.87].^52^ A similar conclusion was drawn by DeFonseka *et al*.^14^ The anti-TNFα therapy also had a favourable impact on VTE risk in the post-hospitalisation period.^9^

The positive effects of anti-TNFα agents have previously been suggested based on laboratory data showing normalisation of thrombolytic characteristics in IBD patients responding to biologics.^60^ These data were supported by a study published by Detrez *et al*.^61^

Based on the efficacy of TNFα inhibitors in inducing and maintaining disease remission, Bernstein *et al.*^4^ presumed reduced VTE risk. In their population-based study, long-term biologic treatment did not significantly alter VTE risk (incidence rate ratio [IRR]: 0.83, CI: 0.40–1.74). CS therapy was associated with an increased risk of VTE either with [IRR: 5.62, CI: 3.24–9.77] or without a concomitant biologic therapy [IRR: 3.03, CI: 2.14–4.30].

Possible explanations were that the protective effect of the biologics was outweighed by their use in patients with more severe disease activity, with a pronounced VTE risk. Delayed initiation of these drugs was also encountered.

Data on whether CS is an independent risk factor of VTE in IBD are inconclusive.^46,47,55,58^ Meta-analytical calculations^59^ have demonstrated that CS therapy results in twice as many events compared with those not receiving CS [OR = 2.202; CI: 1.698–2.856, *p* <0.001].^59^ This effect was considered to be partly independent of IBD activity, as CS also increases coagulability in the non-IBD population.^19,62,63^ It resulted in more events in common clinical presentation of VTE^4,56,64^ in rare cases of porto-mesenteric vein thrombosis [PMVT] [OR = 4.39; CI: 1.27–15.19]^48^ and cerebral venous sinus thrombosis [CVST].^57^ A different effect in the two disease phenotypes has also been reported.^53^

The effect of immunomodulators on VTE risk has not been proven. Indirect data suggest a potential antithrombotic effect of thiopurines based on the inhibition of platelet aggregation^65,66^; clinical evidence is scarce. No significant differences were found in a large cohort comparing VTE risk in patients treated with TNFα inhibitors, thiopurines, methotrexate, or ciclosporin.^50^ A possible beneficial effect of IM therapy was stated in PMVT cases.^48^

More data indicate that IMs do not affect the thromboembolic risk.^14,55,67–70^ Conversely, other data suggest that IM therapy [besides cardiovascular risk, malignancy, and major surgery] may be considered a risk factor for VTE.^51^

There is also a lack of relevant clinical studies on the effect of 5-ASA medication on VTE risk and available data are conflicting. A small study showed reduced spontaneous and thrombin-induced platelet activation independent of disease type and activity.^71^ A randomised controlled trial [RCT] of patients with active UC did not report VTE.^72^ The effect of 5-ASA on VTE was not found in other publications either.^45,55^ Meanwhile, Kim *et al*. published an aHR of 0.15 [CI: 0.09–0.25, p <0.001]^58^ for these drugs in their analysis of the nationwide IBD cohort.

Besides aggressive inflammation control aiming to reduce the risk of complications additionally, thromboprophylaxis is the most effective strategy to prevent VTE. Implementation of current guidelines^3,15^ should become a regular clinical practice.

### 4.1. Strengths and limitations

The main strength of our work is that, to our knowledge, this is the first comprehensive study to investigate the effect of the most commonly used IBD medications on VTE, using rigorous methodology in a relatively large number of IBD patients.

Limitations should be mentioned. Data were mostly from retrospective studies, many of which were monocentric, as there are no randomised controlled trials available on this topic. Data from hospitalised patients show some degree of patient selection bias [more severe disease and increased VTE risk]. Mostly symptomatic patients were evaluated in individual studies, which may underestimate the true prevalence of VTE. Disease characteristics [activity, localisation, and behaviour] are not uniformly and consequently reported; oftentimes medication was used as a proxy for disease activity. The concomitant medication characteristic of this patient population has a major role in accurately defining the effect of each type of anti-inflammatory medication on VTE. Comorbidities affecting VTE risk were not always encountered. There was a lack of reporting of thromboprophylaxis, which significantly influences the occurrence of VTE. Due to these limitations, our meta-analysis provides very low certainty of evidence.

### 4.2. Implications for practice and research

Translating scientific data into daily clinical practice through well-designed models is key.^73,74^

Depending on multiple disease- and patient-specific factors, drug selection in IBD can be challenging. The optimal medical treatment should be tailored to the individual, as better control of disease activity also leads to fewer complications.

On the basis of current data, we highlight the importance of 'steroid-sparing' anti-TNFα therapy, with early initiation of these drugs in patients with active disease flare-up and multiple VTE risk factors. The 'pro-thrombogenic' effect of CS should be considered.

Evidence on the effect of IM and 5-ASA on VTE risk is limited; synthesising data suggests that long-term disease remission maintained with TNFα inhibitors or the mentioned anti-inflammatory medication may result in similar rates of VTE.

Our data point to the future, as prospective, multicentre, large-scale studies are needed to assess the potential association and impact of drugs on VTE in IBD, taking a number of confounding factors into account. Meanwhile, high clinical suspicion, guideline-based thromboprophylaxis, close monitoring, and follow-up of these patients are recommended.

## 5. Conclusions

Available literature and our summary of clinical data indicate that CS may increase the risk of VTE, whereas anti-TNFα agents may reduce the odds of these events compared with CS. We emphasise that in patients with active disease flare-ups with a high VTE risk, anti-TNFα therapy should be preferred over CS treatment.

The recommended thromboprophylaxis should be regularly applied, regardless of the chosen anti-inflammatory medication.

## Supplementary Material

jjad193_suppl_Supplementary_Material

## Data Availability

The datasets used in this study can be found in the full-text articles included in the systematic review and meta-analysis.
