# Supplementary material for: Anti-tumor Necrosis Factor Alpha Versus Corticosteroids: A 3-fold Difference in the Occurrence of Venous Thromboembolism in Inflammatory Bowel Disease－A Systematic Review and Meta-analysis
Source: J Crohns Colitis. 2023 Nov 11;18(5):773–83. doi: 10.1093/ecco-jcc/jjad193 (PMC11140625; doi:10.1093/ecco-jcc/jjad193)
Supplement: jjad193_suppl_Supplementary_Material [file jjad193_suppl_supplementary_material.docx]

**Supplementary Material**

**Content:**

**Supplementary Material Table 1.** Prisma 2020 Checklist^24^

**Supplementary Material** 1. Search key

**Supplementary Material Table 2**. Risk of bias (RoB) assessment based on the Joanna Briggs Critical Appraisal Tool^30^

**Supplementary Material Formula 1.** Sample size calculation^31^

**Supplementary Material Table 3.** Risk of Bias in Non-randomized Studies of Interventions (ROBINS-I)Tool^32^

**Supplementary Material Figure 1.** Forest plot representing the proportion of venous thromboembolic events in inflammatory bowel disease patients treated with tumor necrosis factorα inhibitors

**Supplementary Material Figure 2.** Forest plot showing the proportion of venous thromboembolic events in inflammatory bowel disease patients treated with corticosteroids

**Supplementary Material Figure 3.** Forest plot presentation of the proportion of venous thromboembolism in inflammatory bowel disease patients treated with immunomodulators

**Supplementary Material Figure 4.** Forest plot showing the proportion of venous thromboembolic events in inflammatory bowel disease patients treated with 5-aminosalicylates

**Supplementary Material Figure 5. Forest plot of subgroup analysis** showing the proportion of venous thromboembolic events in Crohn’s disease (CD) or ulcerative colitis (UC) patients treated with A.: anti-tumor necrosis factorα drugs, B.: corticosteroids, C.: immunomodulators, D.: 5-aminosalicylates

**Supplementary Material Figure 6. A., B., C., D.** Funnel plots for visual presentation of bias or systematic heterogeneity across studies

**Supplementary Material Figure 7. A., B., C.** Funel plot representing risk of publication bias

**Supplementary Material** GRADE Assessment^33^

**Supplementary Material Table 4.** Quality of evidence

**Supplementary Material Table 5. Summary of findings table**^34^ presenting the GRADE investigation for the proportion of venous thromboembolic events in the mentioned treatment categories

**Supplementary Material Table 6. Summary of findings table**^34^ presenting the GRADE investigation for the the odds of venous thromboembolic events in the mentioned treatment categories

**Table 1. Prisma 2020 Checklist**^24^**.**

| **Section and Topic** | **Item #** | **Checklist item** | **Location where item is reported** |
| --- | --- | --- | --- |
| **TITLE** | | | page |
| Title | 1 | Identify the report as a systematic review. | 1 |
| **ABSTRACT** | | |  |
| Abstract | 2 | See the PRISMA 2020 for Abstracts checklist. | 4 |
| **INTRODUCTION** | | |  |
| Rationale | 3 | Describe the rationale for the review in the context of existing knowledge. | 6-7 |
| Objectives | 4 | Provide an explicit statement of the objective(s) or question(s) the review addresses. | 6-7 |
| **METHODS** | | |  |
| Eligibility criteria | 5 | Specify the inclusion and exclusion criteria for the review and how studies were grouped for the syntheses. | 7 |
| Information sources | 6 | Specify all databases, registers, websites, organisations, reference lists and other sources searched or consulted to identify studies. Specify the date when each source was last searched or consulted. | 7-8 |
| Search strategy | 7 | Present the full search strategies for all databases, registers and websites, including any filters and limits used. | 7 |
| Selection process | 8 | Specify the methods used to decide whether a study met the inclusion criteria of the review, including how many reviewers screened each record and each report retrieved, whether they worked independently, and if applicable, details of automation tools used in the process. | 8 |
| Data collection process | 9 | Specify the methods used to collect data from reports, including how many reviewers collected data from each report, whether they worked independently, any processes for obtaining or confirming data from study investigators, and if applicable, details of automation tools used in the process. | 9 |
| Data items | 10a | List and define all outcomes for which data were sought. Specify whether all results that were compatible with each outcome domain in each study were sought (e.g. for all measures, time points, analyses), and if not, the methods used to decide which results to collect. | 8 |
|  | 10b | List and define all other variables for which data were sought (e.g. participant and intervention characteristics, funding sources). Describe any assumptions made about any missing or unclear information. | 8 |
| Study risk of bias assessment | 11 | Specify the methods used to assess risk of bias in the included studies, including details of the tool(s) used, how many reviewers assessed each study and whether they worked independently, and if applicable, details of automation tools used in the process. | 9 |
| Effect measures | 12 | Specify for each outcome the effect measure(s) (e.g. risk ratio, mean difference) used in the synthesis or presentation of results. | 10 |
| Synthesis methods | 13a | Describe the processes used to decide which studies were eligible for each synthesis (e.g. tabulating the study intervention characteristics and comparing against the planned groups for each synthesis (item #5)). | 10 |
|  | 13b | Describe any methods required to prepare the data for presentation or synthesis, such as handling of missing summary statistics, or data conversions. | 9 |
|  | 13c | Describe any methods used to tabulate or visually display results of individual studies and syntheses. | 9-10 |
|  | 13d | Describe any methods used to synthesize results and provide a rationale for the choice(s). If meta-analysis was performed, describe the model(s), method(s) to identify the presence and extent of statistical heterogeneity, and software package(s) used. | 10 |
|  | 13e | Describe any methods used to explore possible causes of heterogeneity among study results (e.g. subgroup analysis, meta-regression). | - |
|  | 13f | Describe any sensitivity analyses conducted to assess robustness of the synthesized results. | - |
| Reporting bias assessment | 14 | Describe any methods used to assess risk of bias due to missing results in a synthesis (arising from reporting biases). | - |
| Certainty assessment | 15 | Describe any methods used to assess certainty (or confidence) in the body of evidence for an outcome. | 9 |
| **RESULTS** | | |  |
| Study selection | 16a | Describe the results of the search and selection process, from the number of records identified in the search to the number of studies included in the review, ideally using a flow diagram. | 11 |
|  | 16b | Cite studies that might appear to meet the inclusion criteria, but which were excluded, and explain why they were excluded. | 11-12 |
| Study characteristics | 17 | Cite each included study and present its characteristics. | 11-13 |
| Risk of bias in studies | 18 | Present assessments of risk of bias for each included study. | 20 |
| Results of individual studies | 19 | For all outcomes, present, for each study: (a) summary statistics for each group (where appropriate) and (b) an effect-estimate and its precision (e.g. confidence/credible interval), ideally using structured tables or plots. | 16-20 |
| Results of syntheses | 20a | For each synthesis, briefly summarise the characteristics and risk of bias among contributing studies. | Supl.Tb.2,3 |
|  | 20b | Present results of all statistical syntheses conducted. If meta-analysis was done, present for each the summary estimate and its precision (e.g. confidence/credible interval) and measures of statistical heterogeneity. If comparing groups, describe the direction of the effect. | 16-20 |
|  | 20c | Present results of all investigations of possible causes of heterogeneity among study results. | 21 |
|  | 20d | Present results of all sensitivity analyses conducted to assess the robustness of the synthesized results. | - |
| Reporting biases | 21 | Present assessments of risk of bias due to missing results (arising from reporting biases) for each synthesis assessed. | - |
| Certainty of evidence | 22 | Present assessments of certainty (or confidence) in the body of evidence for each outcome assessed. | 20 |
| **DISCUSSION** | | |  |
| Discussion | 23a | Provide a general interpretation of the results in the context of other evidence. | 21-23 |
|  | 23b | Discuss any limitations of the evidence included in the review. | 23 |
|  | 23c | Discuss any limitations of the review processes used. | 23 |
|  | 23d | Discuss implications of the results for practice, policy, and future research. | 24 |
| **OTHER INFORMATION** | | |  |
| Registration and protocol | 24a | Provide registration information for the review, including register name and registration number, or state that the review was not registered. | 7 |
|  | 24b | Indicate where the review protocol can be accessed, or state that a protocol was not prepared. | 7 |
|  | 24c | Describe and explain any amendments to information provided at registration or in the protocol. | 10 |
| Support | 25 | Describe sources of financial or non-financial support for the review, and the role of the funders or sponsors in the review. | 26 |
| Competing interests | 26 | Declare any competing interests of review authors. | 26 |
| Availability of data, code and other materials | 27 | Report which of the following are publicly available and where they can be found: template data collection forms; data extracted from included studies; data used for all analyses; analytic code; any other materials used in the review. | - |

**1. Search key**

**Search key in Cochrane Central Register of Controlled Trials (CENTRAL), MEDLINE (via PubMed), Scopus, and Web of Science**:

(inflammatory bowel diseases OR IBD OR Crohn Disease OR CD OR Colitis, ulcerative OR CU OR UC) **AND**

(embolism and thrombosis OR embolism OR thromboembolism OR venous thromb* OR venous thromboemb* OR thromboemb* OR VTE OR deep vein thromb* OR DVT OR thromb* OR VT OR pulmonary embolism OR pulmonary thromboemb* OR PE OR PTE OR thromboembolic event* OR venous thromboembolic event*) **AND**

(Antibodies, Monoclonal OR tumor necrosis factor inhibitors OR tumour necrosis factor inhibitors OR tumor necrosis factor OR tumour necrosis factor OR TNF Inhibitor* OR anti-tumor necrosis factor OR anti-tumour necrosis factor OR anti-TNF OR Infliximab OR Adalimumab OR Certolizumab Pegol OR biologic*)

**Search key in Embase:**

((inflammatory bowel diseases OR IBD OR Crohn Disease OR CD OR Colitis, ulcerative OR CU OR UC)) **AND**

((embolism and thrombosis OR embolism OR thromboembolism OR "venous thromb*" OR "venous thromboemb*" OR thromboemb* OR VTE OR "deep vein thromb*" OR DVT OR thromb* OR VT OR pulmonary embolism OR "pulmonary thromboemb*" OR PE OR PTE OR "thromboembolic event*" OR "venous thromboembolic event*")) **AND**

((Antibodies, Monoclonal OR tumor necrosis factor inhibitors OR "tumour necrosis factor inhibitors" OR "tumor necrosis factor" OR "tumour necrosis factor" OR "TNF Inhibitor*" OR "anti-tumor necrosis factor" OR "anti-tumour necrosis factor" OR "anti-TNF" OR Infliximab OR Adalimumab OR Certolizumab Pegol OR biologic*))

**Table 2**. **Risk of bias (RoB) assessment based on the Joanna Briggs Critical Appraisal Tool** – **Checklist for Prevalence Studies**^30^ assessing the proportion of venous thromboembolic events in patients with inflammatory bowel disease

**Interpretation:**

| **Yes** |  |
| --- | --- |
| **No** |  |
| **Unclear** |  |
| **Not applicable** |  |

**Overall risk of bias:**

| **Low risk** |  |
| --- | --- |
| **Moderate risk** |  |
| **High risk** |  |

| **First author**  **and**  **year of publication** | 1. **Was the sample frame appropriate to**   **address the target population?** | 1. **Were study participants recruited in**   **an appropriate way?** | 1. **Was the sample size adequate?** | **4. Where the study subjects and setting**  **described in detail?** | **5. Was data analysis conducted with**  **sufficient coverage of the identified sample?** | **6. Were valid methods used for the**  **identification of the condition?** | **7. Was the condition measured in a standard, reliable way for all participants?** | **8. Was there appropriate statistical analysis?** | **9. Was the response rate adequate and if not,**  **was the low response rate managed appropriately?** | **The overall risk of bias** |
| --- | --- | --- | --- | --- | --- | --- | --- | --- | --- | --- |
| **Alatri et al.,**  **2016** |  |  |  |  |  |  |  |  |  |  |
| **Ando et al., retrosp., 2018** |  |  |  |  |  |  |  |  |  |  |
| **Ando et al.,**  **prosp., 2018** |  |  |  |  |  |  |  |  |  |  |
| **Andrade et al.,**  **2018** |  |  |  |  |  |  |  |  |  |  |
| **Arora et al.,**  **2015** |  |  |  |  |  |  |  |  |  |  |
| **Bernstein et al.,**  **2021** |  |  |  |  |  |  |  |  |  |  |
| **Curtis et al.,**  **2021** |  |  |  |  |  |  |  |  |  |  |
| **deFonseka et al.,**  **2016** |  |  |  |  |  |  |  |  |  |  |
| **Desai et al.,**  **2017** |  |  |  |  |  |  |  |  |  |  |
| **Fujiya et al.,**  **2022** |  |  |  |  |  |  |  |  |  |  |
| **Higgins et al.,**  **2014** |  |  |  |  |  |  |  |  |  |  |
| **Liu et al.,**  **2021** |  |  |  |  |  |  |  |  |  |  |
| **McCurdy et al.,**  **2019** |  |  |  |  |  |  |  |  |  |  |
| **Ohta et al.,**  **2019** |  |  |  |  |  |  |  |  |  |  |
| **Scoville et al.,**  **2014** |  |  |  |  |  |  |  |  |  |  |
| **Shujun et al.,**  **2021** |  |  |  |  |  |  |  |  |  |  |

**Formula 1. Sample size calculation**^31^ – was used to complement data of Joanna Briggs Critical Appraisal Checklist for Prevalence Studies for the proportional analysis of venous thromboembolic events in patients with inflammatory bowel disease (formula by Naing et al., 2006):

**Formula: n = Z^2^P(1−P)/d^2^**

*where:*

*Z* = Z statistic for a level of confidence,

*P* = expected prevalence or proportion (in proportion of one; if 20%, *P* = 0.2),

*d* = precision (in proportion of one; if 5%, *d* = 0.05).

*d =* ½ of P if P<0.1 (10%)

*d =* ½ of P if P>0.9 (90%).

*Z* statistic (*Z*): for the level of confidence of 95%, which is conventional, *Z* value is 1.96.

Example: proportion of venous thromboembolic events in inflammatory bowel disease.

P = 0,05 (the proportion of venous thromboembolic events in inflammatory bowel disease).

d = 0,025 (precision is ½ P if the proportion of the disease – inflammatory bowel disease – is <10%).

**n = (1.96)^2^*0.05*(1-0.05)/ (0.025)^2^ ; n = 291,96.**

n (291,96) was our reference value to appreciate the adequate sample sizes in the process of determining the risk of bias for the proportion of venous thromboembolic events in patients with inflammatory bowel disease.

**Table 3. Risk of Bias in Non-randomized Studies of Interventions (ROBINS-I) Tool**^32^ - used to evaluate the possible association of medications and venous thromboembolic events in patients with inflammatory bowel disease

**Interpretation:**

| **Low** |  |
| --- | --- |
| **Intermediate** |  |
| **Serious** |  |
| **No information** |  |

**Overall risk of bias:**

| **Low risk** |  |
| --- | --- |
| **Moderate risk** |  |
| **High risk** |  |

| **Study** | | **Pre-intervention** | | | **At**  **intervention** | **Post-intervention** | | | | | | **Overall risk of bias** |
| --- | --- | --- | --- | --- | --- | --- | --- | --- | --- | --- | --- | --- |
| **First author and**  **publication year** | **Bias due**  **to confounding** | | **Bias in selection of study participants** | **Bias in classification of interventions** | | | **Bias due to deviations from the intended interventions** | **Bias due to missing data** | **Bias in measure-**  **ment of**  **outcome** | **Bias in selection of the reported result** | |  |
| **Alatri et al.,**  **2016** |  | |  |  | | |  |  |  |  |  | |
| **Ando et al.,retrospective,**  **2018** |  | |  |  | | |  |  |  |  |  | |
| **Ando et al., prospective,**  **2018** |  | |  |  | | |  |  |  |  |  | |
| **Andrade et al.,**  **2018** |  | |  |  | | |  |  |  |  |  | |
| **Arora et al.,**  **2015** |  | |  |  | | |  |  |  |  |  | |
| **Ananthakrishnan et al.,**  **2014** |  | |  |  | | |  |  |  |  |  | |
| **Bernstein et al.,**  **2021** |  | |  |  | | |  |  |  |  |  | |
| **Curtis et al.,**  **2021** |  | |  |  | | |  |  |  |  |  | |
| **deFonseka et al.,**  **2016** |  | |  |  | | |  |  |  |  |  | |
| **Desai et al.,**  **2017** |  | |  |  | | |  |  |  |  |  | |
| **Fujiya et al.,**  **2022** |  | |  |  | | |  |  |  |  |  | |
| **Higgins et al.,**  **2014** |  | |  |  | | |  |  |  |  |  | |
| **Kim et al.,**  **2021** |  | |  |  | | |  |  |  |  |  | |
| **Liu et al.,**  **2021** |  | |  |  | | |  |  |  |  |  | |
| **McCurdy et al.,**  **2019** |  | |  |  | | |  |  |  |  |  | |
| **Ohta et al.,**  **2019** |  | |  |  | | |  |  |  |  |  | |
| **Scoville et al.,**  **2014** |  | |  |  | | |  |  |  |  |  | |
| **Shujun et al.,**  **2021** |  | |  |  | | |  |  |  |  |  | |

**Figure 1. Forest plot** **representing the proportion of venous thromboembolic events in inflammatory bowel disease patients treated with tumor necrosis factorα inhibitors**


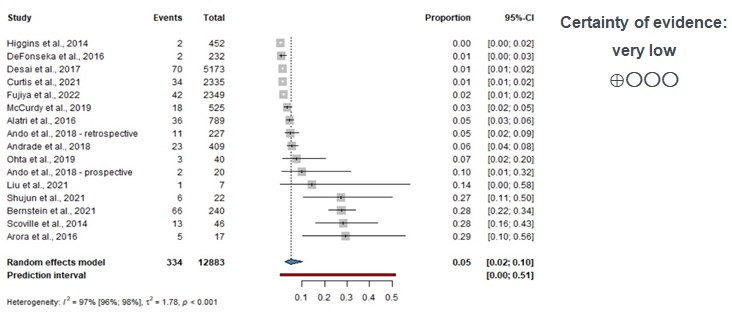


**Explanations, abbreviations**: Events: venous thromboembolic events; Total: number of inflammatory bowel disease patients treated with tumor necrosis factorα inhibitors; CI: confidence interval.

**Figure 2. Forest plot showing the proportion of venous thromboembolic events in inflammatory bowel disease patients treated with corticosteroids**


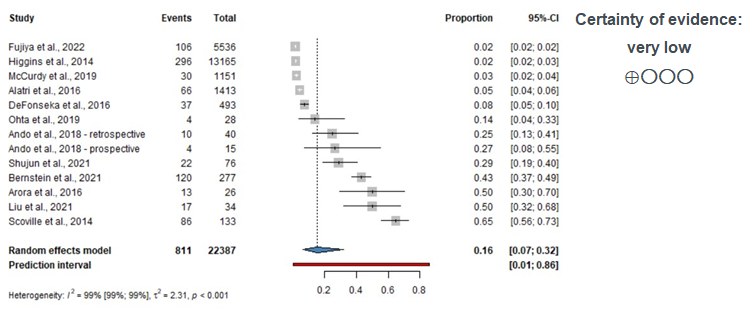


**Explanations, abbreviations**: Events: venous thromboembolic events; Total: number of inflammatory bowel disease patients treated with corticosteroids; CI: confidence interval.

**Figure 3. Forest plot presentation of the proportion of venous thromboembolism in inflammatory bowel disease patients on immunomodulators**


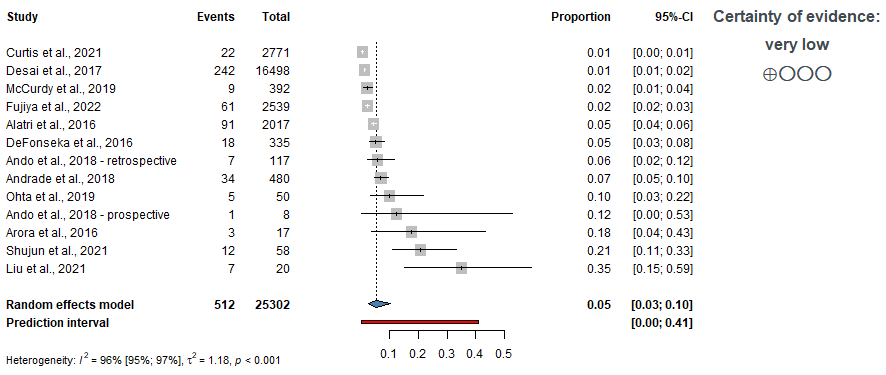


**Explanations, abbreviations**: Events: venous thromboembolic events; Total: number of inflammatory bowel disease patients treated with immunomodulators; CI: confidence interval.

**Figure 4. Forest plot showing the proportion of venous thromboembolic events in inflammatory bowel disease patients on 5-aminosalicylates**


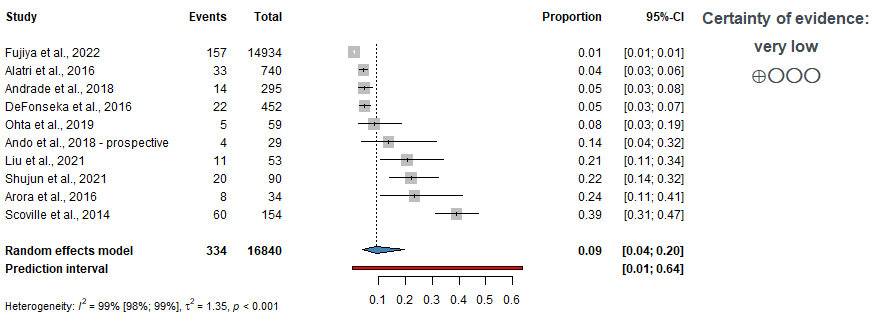


**Explanations, abbreviations**: Events: venous thromboembolic events; Total: number of inflammatory bowel disease patients treated with 5-aminosalicylates; CI: confidence interval.

**Figure 5. Forest plot of subgroup analysis** **showing the proportion of venous thromboembolic events in Crohn’s disease (CD) or ulcerative colitis (UC) patients treated with A.: anti-tumor necrosis factorα drugs, B.: corticosteroids, C.: immunomodulators, D.: 5-aminosalicylates**.

**5A.**


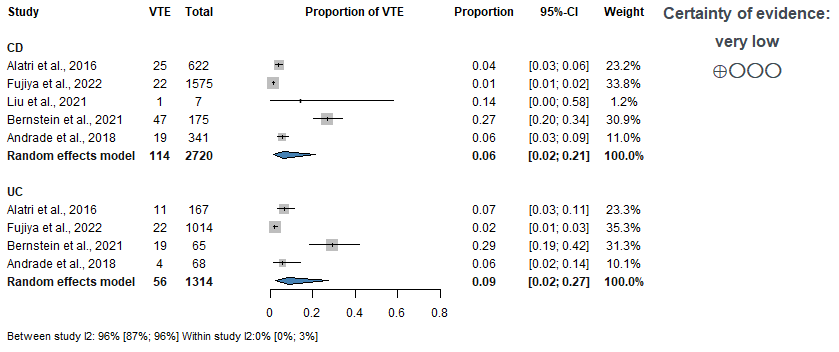


**5B.**


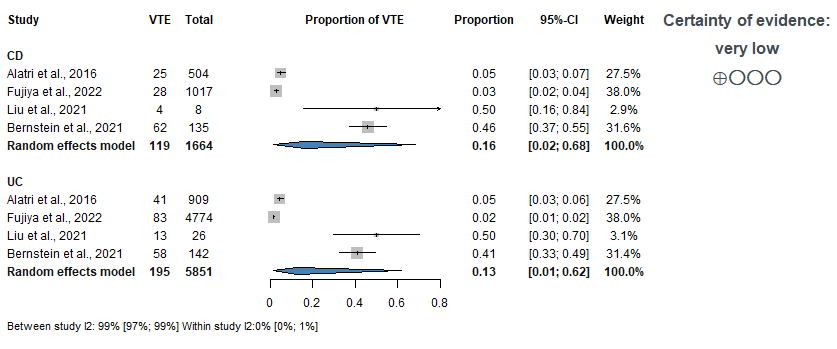


**5C.**


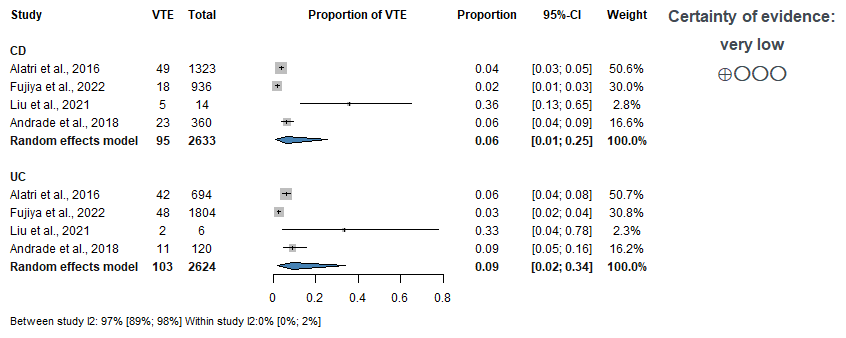


**5D.**


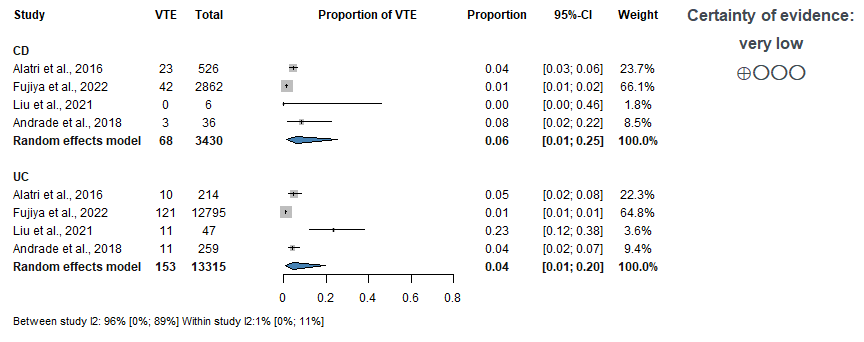


**Abbreviations**: VTE: venous thromboembolic events; Total: number of inflammatory bowel disease patients treated with:

A: tumor necrosis factorα inhibitors; B: corticosteroids; C: immunomodulators; D: 5-aminosalicylates; CI: confidence interval; CD: Crohn’s disease; UC: ulcerative colitis.

**Figure 6. A. Funnel plot – for visual presentation of bias or systematic heterogeneity across studies reporting venous thromboembolism in patients on anti-tumor necrosis factorα therapy**


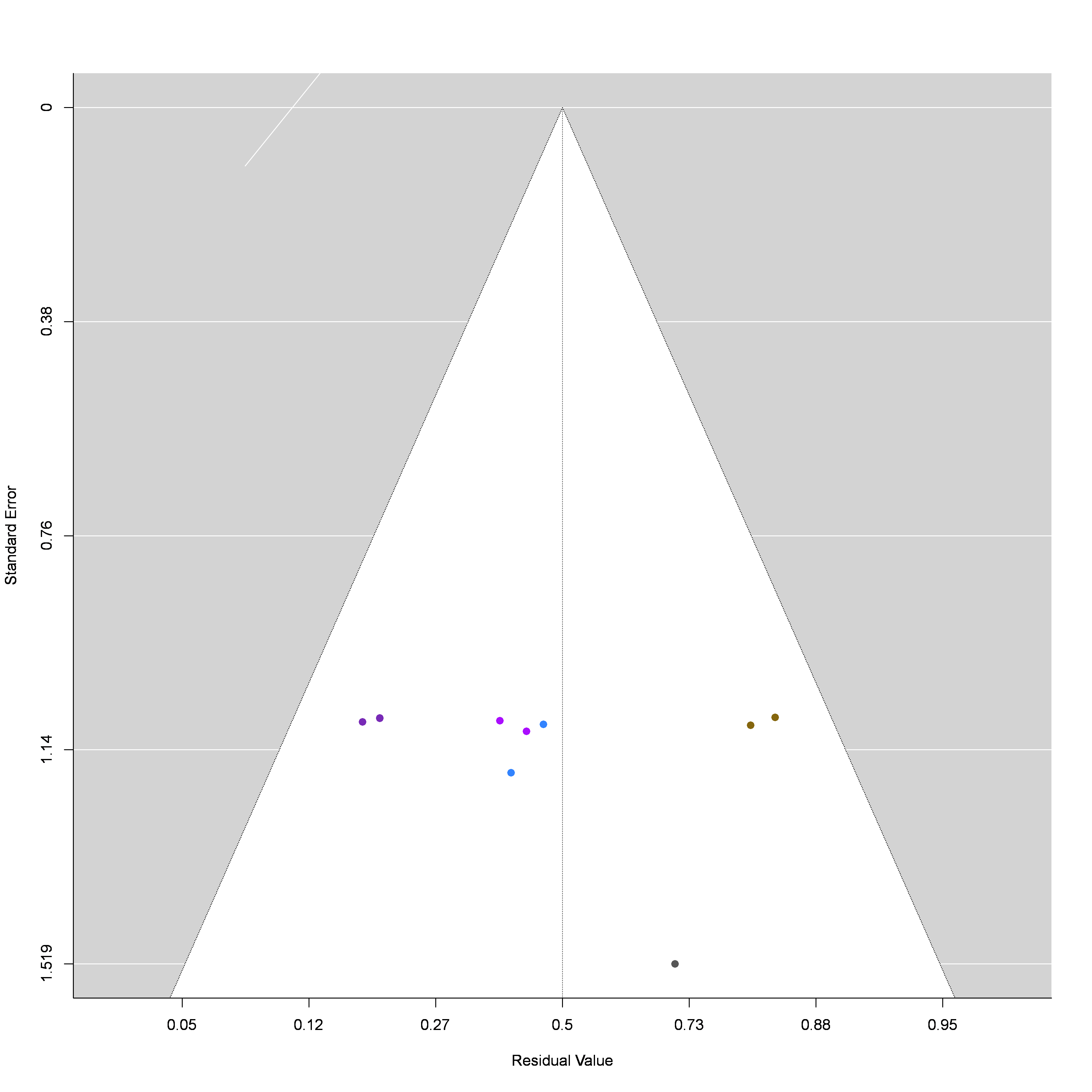


**Figure 6. B. Funnel plot – for visual presentation of bias or systematic heterogeneity across studies reporting venous thromboembolic events related to corticosteroid treatment**

**
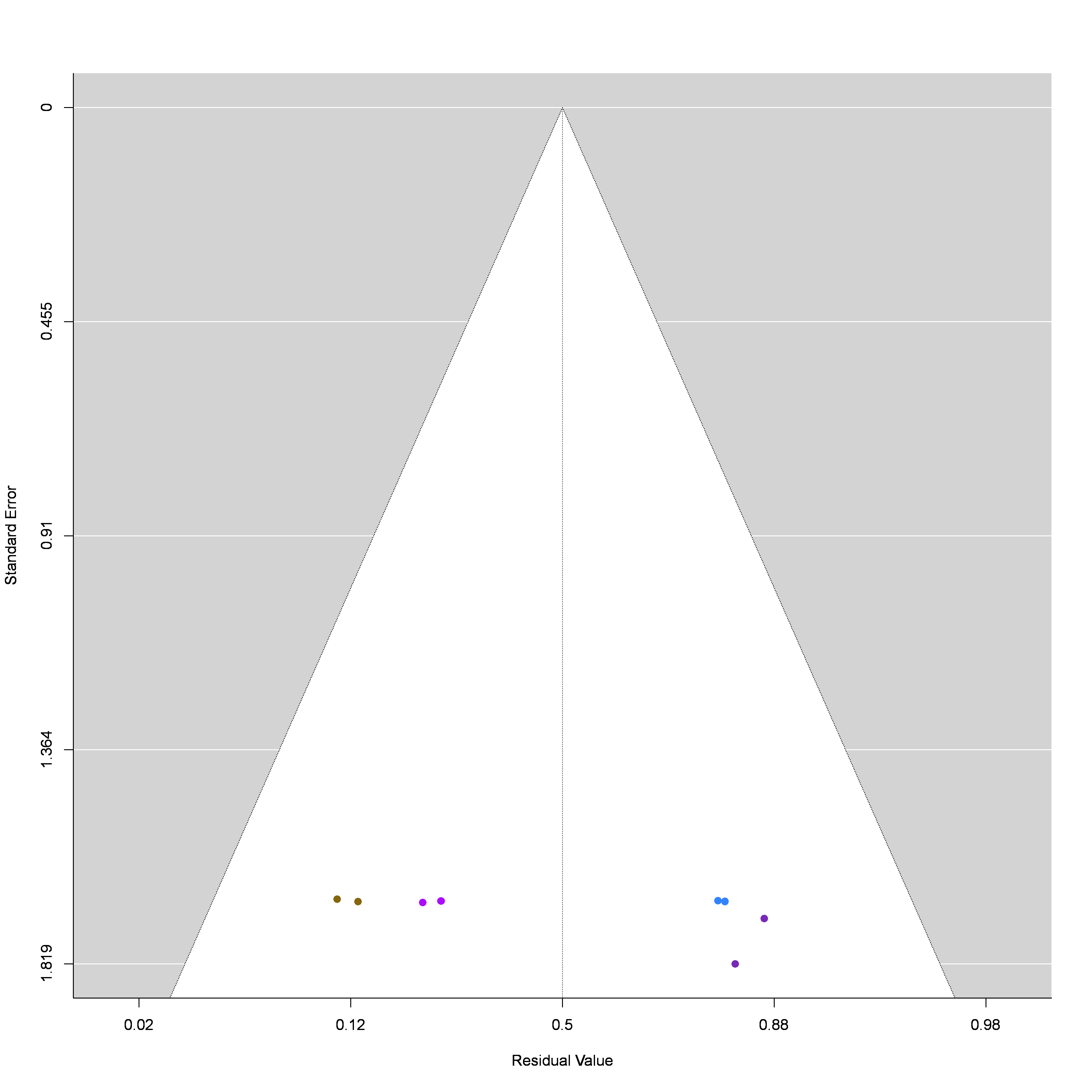
**

**Figure 6. C. Funnel plot – for visual presentation of bias or systematic heterogeneity across studies conserning venous thromboembolism in patients treated with immunomodulators**


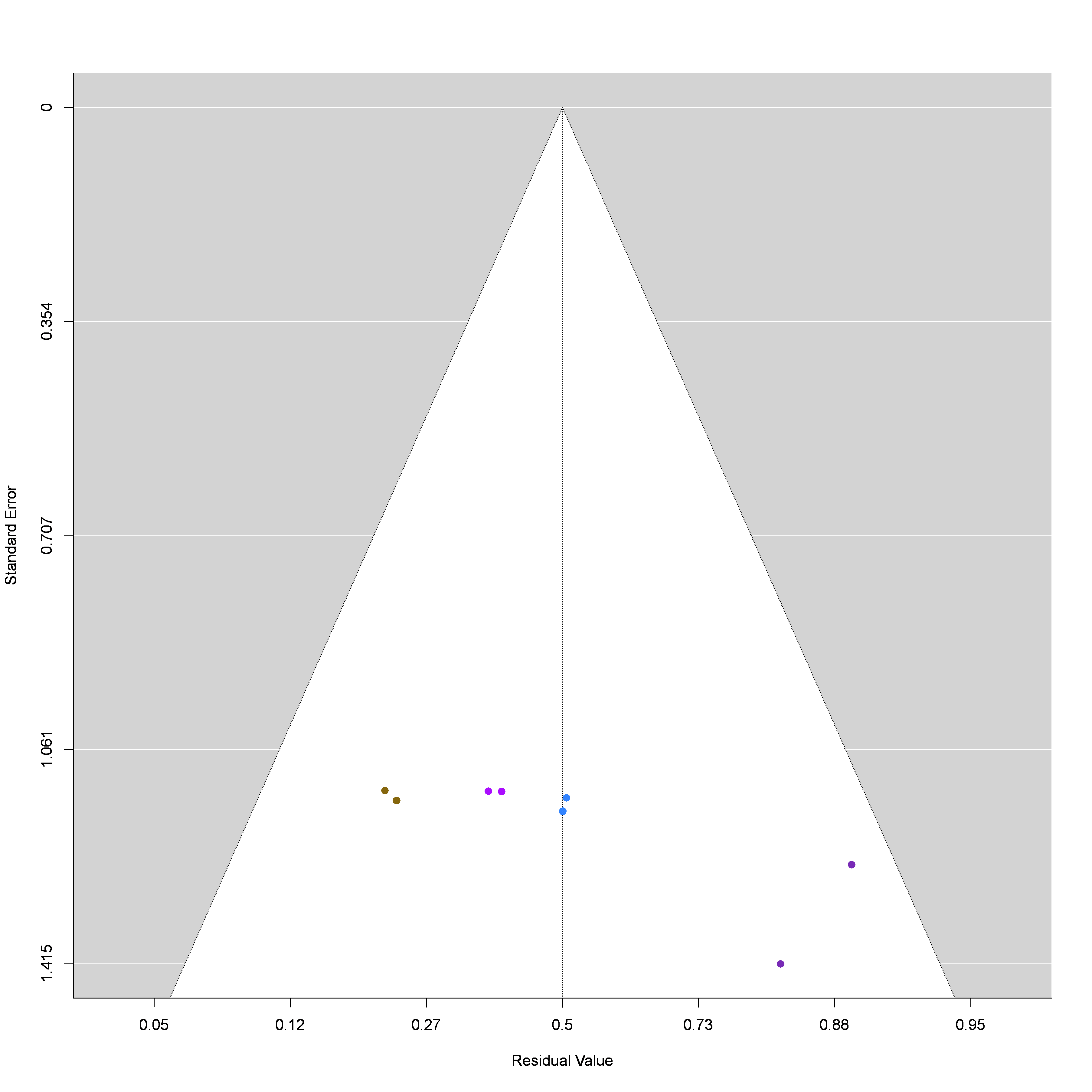


**Figure 6. D. Funnel plot – for visual presentation of bias or systematic heterogeneity across studies in cases of patients treated with 5-aminosalicylates having venous thromboembolism**


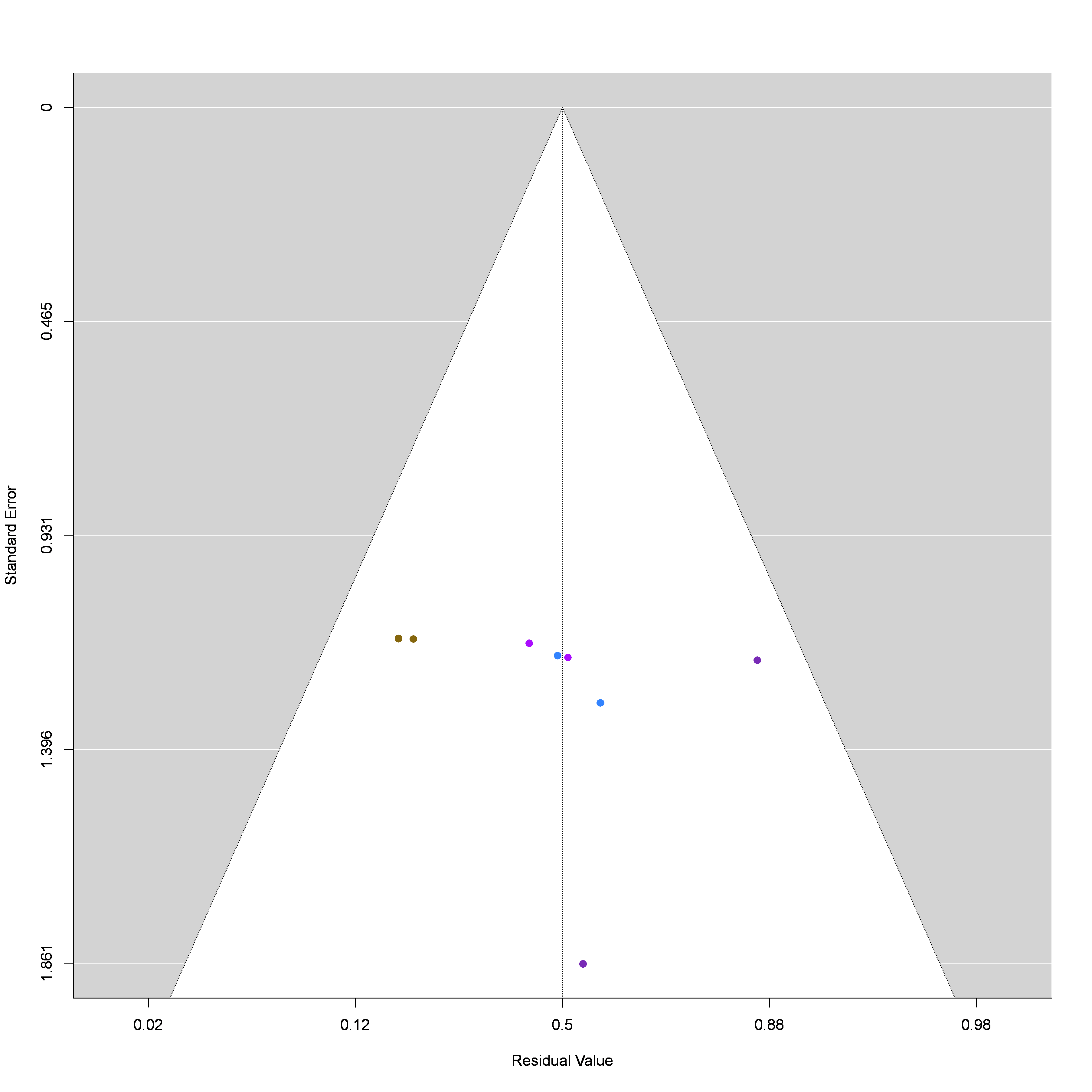


**Figure 7. A. Funnel plot** **of included studies assessing the role of anti-tumor necrosis factorα versus corticosteroid therapy on venous thromboembolic events in inflammatory bowel disease patients – detection of publication bias or systematic heterogeneity.**

Based on the odds ratio of corticosteroids versus anti-tumor necrosis factorα drugs, the asymmetry of the funnel plot suggests the possibility of systematic heterogeneity (clinical differences between studies) rather than publication bias.


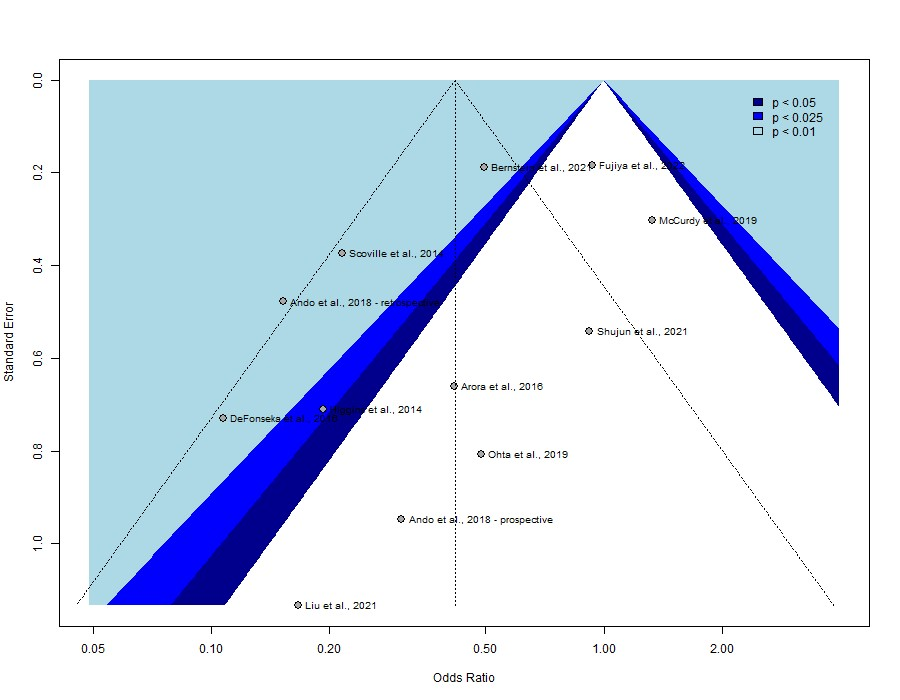


**Figure 7.B. Funnel plot of included studies assessing the role of anti-tumor necrosis factorα and immunomodulator drugs on venous thromboembolic events in inflammatory bowel disease patients – detection of publication bias or systematic heterogeneity**.

Taking into account the odds ratio of immunomodulators versus anti-tumor necrosis factorα drugs, the symmetry of the funnel plot suggests a low risk of publication bias.


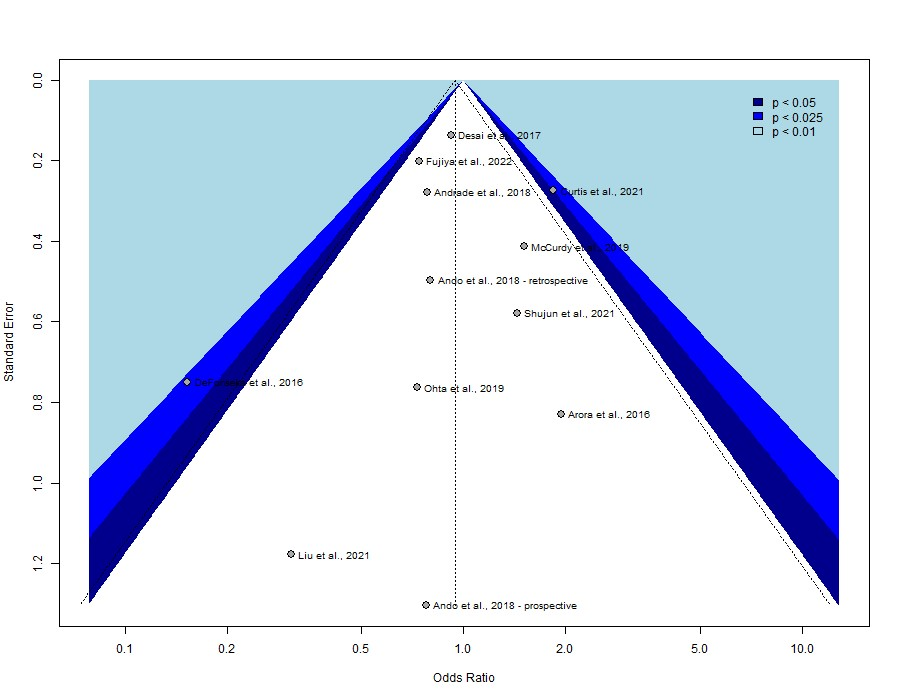


**Figure 7. C. Funnel plot of included studies assessing the role of biologics and 5-aminosalicylates on venous thromboembolic events in inflammatory bowel disease patients – detection of publication bias or systematic heterogeneity**.

Considering the odds ratio of 5-aminosalicylates versus anti-tumor necrosis factorα drugs, the symmetry of the funnel plot suggests a low risk of publication bias.

The article by Fujiya et al. might be an outlier, as the authors stated, that not all of the available data are reported in their study.

**
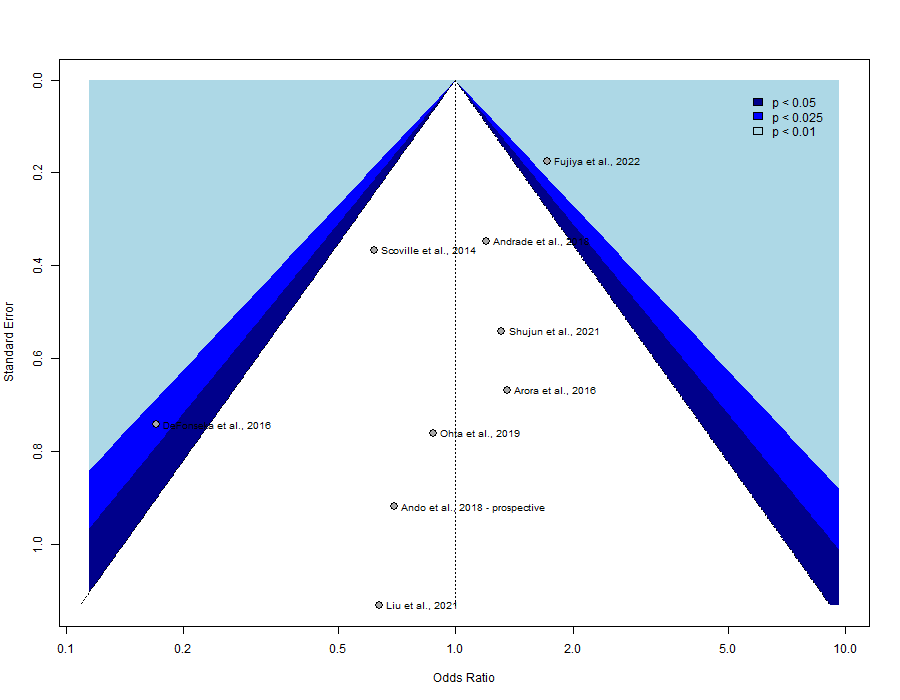
**

**GRADE Assessment^33^**

**Detailed Predefined Criteria to establish the Summary of findings table:**

based on GRADE suggestion of the initial separate consideration of five categories and the reasons for downgrading the quality of evidence, the overall rating of the evidence was considered to be very low.

**The study design** (observational studies) provides a low quality of evidence.

Among **study limitations,** the followings were considered:

- **risk of bias** - primarily due to failure to adequately control for confounding factors;
- **inconsistency of results** - may be partially explained by differences in IBD patient population characteristics between included studies; based on the used statistics, the considerable *I*^2^ statistic and *τ*^2^ (tau square) values point towards inconsistency;
- **indirectness** - the differences in IBD patient population were considered regarding applicability of results;
- **imprecision** - due to the relatively low number of IBD patients and VTE events;
- **publication bias –** as a consequence of observational study design and data collection from medical records in certain articles, the small sample size of several studies, and well as the asymmetry of the funnel plots.

**Table 4. Quality of evidence**

**Summary of Findings table (SoF)**^34^**:** presenting the GRADE investigation of the odds of venous thromboembolic events in patients treated with anti-tumor necrosis factorα inhibitors and conventional anti-inflammatory therapy (corticosteroids, immunomodulators or 5-aminosalicylates).

**Does anti-tumor necrosis factorα therapy decrease the odds of venous thromboembolic events compared to conventional anti-inflammatory medication?**

| **Outcome** | **Anticipated absolute effects** (95% CI) | |  |  |  |  |
| --- | --- | --- | --- | --- | --- | --- |
|  | **Risk with anti-TNFα** | **Risk with cortico-steroids** | **Relative effect**  **(95% CI)** | **Nr. of participants (studies)** | **Certainty of the evidence**  **(GRADE)** | **Comments** |
| **VTE** | 15 per 1000  (9 to 25) | 36 per 1000 | **OR=0.42**  (0.25-0.71) | 25.151  (12 studies) | **⨁◯◯◯ Very low^a,b,c,d^** | Anti-TNF α therapy is associated with decreased odds of VTE compared to cortiosteroids. |
| **Outcome** | **Risk with anti-TNFα** | **Risk with**  **immuno-modulators** | **Relative effect**  **(95% CI)** | **Nr. of participants (studies)** | **Certainty of evidence**  **(GRADE)** | **Comments** |
| **VTE** | 17 per 1000  (14 to 24) | 18 per 1000 | **OR=0.94**  (0.67-1.33) | 34.641  (12 studies) | **⨁◯◯◯ Very low^a,b,c,d^** | There’s no difference of the odds of VTE in patients treated with anti-TNFα therapy or immunomodulators. |
| **Outcome** | **Risk with anti-TNFα** | **Risk with**  **5-aminosali-**  **cylates** | **Relative effect**  **(95% CI)** | **Nr. of participants (studies)** | **Certainty of evidence**  **(GRADE)** | **Comments** |
| **VTE** | 19 per 1000  (11 to 30) | 19 per 1000 | **OR=1.00**  (0.61-1.62) | 19.242  (9 studies) | **⨁◯◯◯ Very low^a,b,c,d^** | Patients treated with anti-TNFα drugs or 5ASA harbor equal odds of VTE. |

**Abbreviation:** VTE: venous thromboembolic events; CI: confidence interval, TNF: tumor necrosis factor, OR: odds ratio; Nr.: number.

**GRADE Working Group grades of evidence:**

**High certainty:** we are very confident that the true effect lies close to that of the estimate of the effect.

**Moderate certainty:** we are moderately confident in the effect estimate: the true effect is likely to be close to the estimate of the effect, but there is a possibility that it is substantially different.

**Low certainty:** our confidence in the effect estimate is limited: the true effect may be substantially different from the estimate of the effect.

**Very low certainty:** we have very little confidence in the effect estimate: the true effect is likely to be substantially different from the estimate of effect.

**Explanations:**

a. Risk of bias is considered to be serious primarily based on failure to adequately control for all of the confounding factors.

b. Inconsistency is serious because there is a large difference in the odds ratio as well as the considerable *I*^2^ statistic, which quantifies the proportion of the variation in point estimates due to among-study differences.

c. Indirectness is serious based on differences in the IBD patient population.

d. Imprecision is serious, because of the small number of patients and events.

**Table 5. Summary of findings table**^34^ presenting the GRADE investigation for the proportion of venous thromboembolic events in the mentioned treatment categories.

| **Nr. of**  **studies** | **Certainty assessment** | | | | | | **Effect** | | | **Certainty** | **Importance** |
| --- | --- | --- | --- | --- | --- | --- | --- | --- | --- | --- | --- |
|  | **Study design** | **Risk of bias** | **Inconsis- tency** | **Indirect-ness** | **Impre-cision** | **Other conside-**  **rations** | **Nr. of events** | **Nr. of indivi-duals** | **Rate (95% CI)** |  |  |
| **Anti-TNFα drugs** | | | | | | | | | | | |
| 16 | observa-tional studies | serious^a^ | serious^b^ | serious^c^ | serious^d^ | ^e,f,g,h^ | 334 | 12.883 | **0.05**  **(0.02-0.10)** | **⨁◯◯◯ Very low** | IMPORTANT |
| **Corticosteroids** | | | | | | | | | | | |
| 13 | observa-tional studies | serious^a^ | serious^b^ | serious^c^ | serious^d^ | ^e,f,g,h^ | 811 | 22.387 | **0.16**  **(0.07-0.32)** | **⨁◯◯◯ Very low** | IMPORTANT |
| **Immunomodulators** | | | | | | | | | | | |
| 13 | observa-tional studies | serious^a^ | serious^b^ | serious^c^ | serious^d^ | ^e,f,g,h^ | 512 | 25.302 | **0.05**  **(0.03-0.10)** | **⨁◯◯◯ Very low** | IMPORTANT |
| **5-aminosalicylates** | | | | | | | | | | | |
| 10 | observa-tional studies | serious^a^ | serious^b^ | serious^c^ | serious^d^ | ^e,f,g,h^ | 334 | 16.840 | **0.09**  **(0.04-0.20)** | **⨁◯◯◯ Very low** | IMPORTANT |

**Abbreviation:** Nr.: number; anti-TNF: anti-tumor necrosis factor, CI: confidence interval.

**Explanations:**

a. Risk of bias is considered to be serious primarily based on failure to adequately control for all of the confounding factors.

b. Inconsistency is serious because there is a large difference in the odds ratio as well as the considerable *I*^2^ statistic, which quantifies the proportion of the variation in point estimates due to among-study differences.

c. Indirectness is serious based on differences in the IBD patient population.

d. Imprecision is serious, because of the relatively small number of patients and events.

e. Based on the asymmetry of the funnel plot, the observational design of included studies as well as the small size of many of the studies, publication bias is very probable.

f. According to the summary of data, there’s no large effect of the interventions.

g. There are a plethora of confounding factors – reporting of these are deficient, imprecise.

h. Taking into account the characteristic dosing of the anti-TNFα and corticosteroids, a dose-response effect is not probable.

**Table 6. Summary of findings table**^34^ presenting the GRADE investigation for the the odds of venous thromboembolic events in the mentioned treatment categories.

| **Nr. of**  **studies** | **Certainty assessment** | | | | | | | | | | | **Effect** | | | | **Certainty** | **Importance** |
| --- | --- | --- | --- | --- | --- | --- | --- | --- | --- | --- | --- | --- | --- | --- | --- | --- | --- |
|  | **Study design** | | **Risk of bias** | | **Inconsis- tency** | | **Indirect-ness** | | **Impre-cision** | | **Other consi-dera-tions** | **Nr. of events** | **Nr. of indivi-duals** | | **OR (95% CI)** |  |  |
| **Anti-TNFα drugs versus corticosteroids** | | | | | | | | | | | | | | | | | |
| 12 | observa-tional studies | serious^a^ | | serious^b^ | | serious^c^ | | serious^d^ | | ^e,f,g,h^ | | 171 vs.  745 | 4.177 vs.  20.974 | **0.42**  **(0.25-0.71)** | | **⨁◯◯◯ Very low** | IMPORTANT |
| **Anti-TNFα drugs vs. immunomodators** | | | | | | | | | | | | | | | | | |
| 13 | observa-tional studies | serious^a^ | | serious^b^ | | serious^c^ | | serious^d^ | | ^e,f,g,h^ | | 217 vs.  421 | 11.356 vs.  23.285 | **0.94**  **(0.67-1.33)** | | **⨁◯◯◯ Very low** | IMPORTANT |
| **Anti-TNFα drugs vs. 5-aminosalicylates** | | | | | | | | | | | | | | | | | |
| 9 | observa-tional studies | seri-ous^a^ | | serious^b^ | | serious^c^ | | serious^d^ | | ^e,f,g,h^ | | 97 vs.  301 | 3.142 vs.  16.100 | **1.00**  **(0.61-1.62)** | | **⨁◯◯◯ Very low** | IMPORTANT |

**Abreviation:** Nr.: number; anti-TNF: anti-tumor necrosis factor; OR: odds ratio; CI: confidence interval; vs.: versus.

**Explanations:**

a. Risk of bias is considered to be serious primarily based on failure to adequately control for all of the confounding factors.

b. Inconsistency is serious because there is a large difference of the odds ratio as well as the considerable *I*^2^ statistic, which quantifies the proportion of the variation in point estimates due to among-study differences.

c. Indirectness is serious based on differencies of the IBD patient population.

d. Imprecision is serious, because of the relatively small number of patients and events.

e. Based on the asymetry of the funnel plot, the observational design of included studies as well as small size of many of the studies, publication bias is very probable.

f. According to the summary of data, there’s no large effect of the interventions.

g. There are a plethora of confounding factors – reporting of these are deficient, imprecise.

h. Taking into account the characteristic dosing of the anti-TNF α and corticosteroids, a dose-response effect is not probable.
